# Adoption of Human Personality Development Theory Combined With Deep Neural Network in Entrepreneurship Education of College Students

**DOI:** 10.3389/fpsyg.2020.01346

**Published:** 2020-07-08

**Authors:** Zhen Chen, Xiaoxuan Yu

**Affiliations:** School of Humanities and Law, Northeastern University, Shenyang, China

**Keywords:** deep learning neural network, entrepreneurial intention, college students’ entrepreneurial mental resilience, human personality development theory, entrepreneurial tenacity

## Abstract

In this research, the probability matrix factorization (PMF) algorithm was introduced to optimize the deep neural network algorithm model with the purpose of studying the application value of personality development theory and deep learning neural network in college students’ entrepreneurship psychological education courses. Based on the personality development theory, a recommendation algorithm system for entrepreneurial projects under optimized deep neural network was established. A total of 518 college students from several universities were divided into an experimental group and a control group, with 259 students in each group. In addition to the normal courses of entrepreneurship psychology education, students in the experimental group were taught the entrepreneurship project recommendation system based on the optimized deep neural network designed in this research, while students in the control group were taught entrepreneurship psychology education normally. The intervention effect before and after entrepreneurship education was evaluated by the questionnaire of college students’ entrepreneurial intention and college students’ entrepreneurial mental resilience scale. The results demonstrate that the system recall rate and accuracy based on the algorithm in this research have been significantly higher than that of PMF algorithm and deep belief network (DBN) algorithm, and the difference is statistically significant (*p* < 0.05); the mean square error (MSE) of the proposed algorithm is significantly smaller than that of PMF algorithm and DBN algorithm, and the difference is statistically significant (*p* < 0.05); the improvement of entrepreneurial toughness, entrepreneurial strength, optimism, entrepreneurial possibility, and behavioral tendency of the experimental group after the test was significantly higher than that of the control group (*p* < 0.05). Therefore, compared with traditional algorithms, the proposed method for entrepreneurial projects based on the theory of personality development and the optimized deep neural network shows better performance, and it can effectively improve the entrepreneurial intention and psychological resilience of college students.

## Introduction

With the development of the socialist market economy, college students are under greater pressure with regards to employment. Self-employment has become a way for college graduates to find employment under the current social background. However, many college students have not truly entered society and developed inadequate entrepreneurial psychological qualities, thereby leading to the failure of college students’ entrepreneurship and low success rate (Kim et al., 2020; Manjusha and Green, 2020; Younis and Katsioloudes, 2020). Therefore, in recent years, various colleges and universities also actively carry out entrepreneurship education courses, so as to enhance the entrepreneurial awareness and ability of college students, improve their entrepreneurial psychology, and establish the entrepreneurial spirit of self-improvement (Boysen et al., 2020). However, due to the late start of entrepreneurship psychological education in Chinese colleges and universities, limited economic conditions, and insufficient emphasis on entrepreneurship education awareness, many colleges and universities only offer one or two courses perfunctorily, and the education system is not systematic (Wu et al., 2019b). Moreover, the courses and research cannot be well correlated with entrepreneurial practice, and pure theoretical nature does not effectively help entrepreneurial college students (Bazan et al., 2020; Karunia et al., 2020). At present, many students in the school actually have the entrepreneurial tendency and psychology, but they lack the entrepreneurial experience. In addition, the psychological education of entrepreneurship in colleges and universities is a mere formality, which is unable to solve their problems (Ahmed et al., 2020). Therefore, it becomes crucial to create a good entrepreneurial psychological education environment, to implement college students’ entrepreneurial psychological education, and to really help entrepreneurial college students (Wu et al., 2019a).

In recent years, deep learning has been widely applied in the fields of computer vision and speech recognition, and has achieved great success (Varadarajan et al., 2020). The deep neural network technology can also be used in the recommendation system, such as the music recommendation function based on the convolutional neural network, which can extract the tonal features of music; the personalized recommendation technology based on the reverse artificial neural network can recode the feature into the low-dimensional vector and calculate the prediction score using the implicit feature; the recommendation system based on the restricted Boltzmann machine can use the visual layer to automatically decode and encode the new scoring data of scored items, and predict the scores of unscored items (Healy et al., 2019; Raissi et al., 2019; Tuli et al., 2020). The application of deep neural network greatly improves the computational accuracy of the current recommendation system compared with the past. The personality development theory was put forward by American psychologist Erikson, who pointed out that human personality development includes the harmonious development of body and mind and the coordination and unity of individuals and society, and runs through the whole life journey (Chen, 2019; Woods et al., 2019). This theory is also an important theoretical basis for entrepreneurship education of college students. When formulating entrepreneurship education programs and teaching plans in colleges and universities, students’ individual development should be fully considered, and students should be taught according to their aptitude, so as to encourage students to actively participate in entrepreneurship practice activities, which is very important to support the entrepreneurial psychology and behavior of college students in the future (Deventer et al., 2019; Ryan et al., 2019).

To sum up, there is much research on the recommendation system of deep neural network, but there are few recommendation systems for the entrepreneurial psychological education of college students. Based on this, 518 students from several universities were selected to be divided into experimental group and control group, with 264 students in each group. In addition to the normal courses of entrepreneurship education, students in the experimental group were supplemented by the entrepreneurship project recommendation systems based on the optimized deep neural network designed in this research. Students in the control group were given entrepreneurship education courses normally (Qian et al., 2018). Through the simulation experiment and the comparative analysis of the change of students’ entrepreneurial intention and entrepreneurial mental resilience before and after the education, the application value of the personality development theory and the deep learning neural network in the courses of college students’ entrepreneurial psychological education were comprehensively evaluated (Wu and Song, 2019).

## Literature Review

In order to improve the entrepreneurial enthusiasm and entrepreneurial ability of college students, many scholars have conducted analyses on the entrepreneurship education of college students. Oguntimehin (2018) investigated the relationship between entrepreneurship education and career entrepreneurship intention of students in Ogun National University and was found that entrepreneurship education significantly affected students’ entrepreneurial intention, so entrepreneurship education should focus on encouraging students to participate in more classroom interaction and further enhance motivation. Cheng et al. (2018) analyzed the existing problems of engineering education in Chinese universities through literature research and questionnaire survey, and on the basis of the previous research, proposed a new engineering student training program combining entrepreneurship education with the specific strategy of cultivating the entrepreneurial ability of engineering talents. Udoye and Mba (2018) adopted a descriptive survey to explore strategies to promote entrepreneurship education for college students in the delta region. Entrepreneurship education would provide life and vocational skills, expand personal potential, enhance self-sufficiency, and improve the quality of life. Moreover, in any institution that strived to develop entrepreneurship, every student should be involved, regardless of their field of expertise. Some scholars have also tried to apply various methods and systems to improve the effectiveness of entrepreneurship education courses for college students. Wu et al. (2019b) used Classroom response systems (CRS) to help 22 graduate students who attended the entrepreneurship management course, and found that mobile CRS technology was an effective tool to promote the interaction between learners and content, increase students’ participation in the acquisition of entrepreneurial knowledge, and enhance students’ motivation to improve their entrepreneurial ability. Dou et al. (2019) systematically contacted the student samples of entrepreneurship education courses through the pilot entrepreneurship program, which provided relevant inspirations for the design of entrepreneurship education and enabled entrepreneurship education to have a more positive impact on entrepreneurship attitudes or activities. Wu et al. (2018) applied the web-based PowToon tool to college students’ entrepreneurship education courses, and allowed teachers and students to quickly and easily create animated demonstrations; it turned out that the videos helped the teams better communicate their business ideas to investors in a thoughtful way, thus changing the traditional model of entrepreneurship education from classroom lectures to case studies to group discussions.

To sum up, the current research on improving the entrepreneurial ability of college students focuses on assisting the development of entrepreneurship education courses with the help of various systems and platforms, which ultimately affects the entrepreneurial intention and personal ability of college students. Therefore, the deep learning network is used to build a targeted entrepreneurship project recommendation system for entrepreneurship education courses to help college students to choose and master entrepreneurship.

## Methodology

### Research Object and Current Situation of Entrepreneurial Psychology

In this study, 518 college students were randomly selected from a number of universities as research objects and were divided into an experimental group and a control group by blind selection, with 259 students in each group. In addition to the normal courses of entrepreneurial psychology education, students in the experimental group were helped to make clear their choice of entrepreneurship by applying the recommendation system of entrepreneurial projects based on the optimized deep neural network designed in this research, while students in the control group were given normal courses of entrepreneurial psychology education (Obschonka et al., 2018). The study took place over 1 month.

**FIGURE 1:**
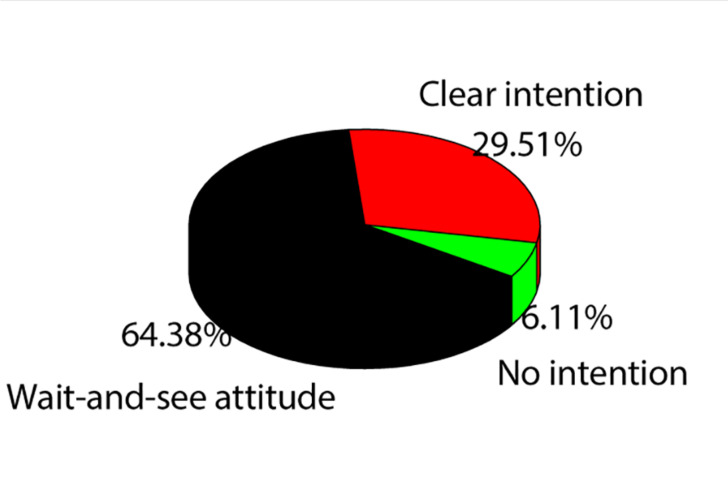
Entrepreneurial psychology status of all subjects.

As shown in Figure 1, among all the selected college students, only 153 have clear entrepreneurial intention, accounting for 29.51%. There are 333 people with a wait-and-see attitude, accounting for 64.38%. Eight people, or 6.11 percent, have little thought of starting a business. For college students, most of them have an uncertain attitude toward entrepreneurship, and only a few of them have a positive attitude toward entrepreneurship in the future. Accordingly, the implementation of entrepreneurship education in colleges and universities is of great significance.

### Framework of the Traditional Deep Learning Recommendation Algorithm

**FIGURE 2:**
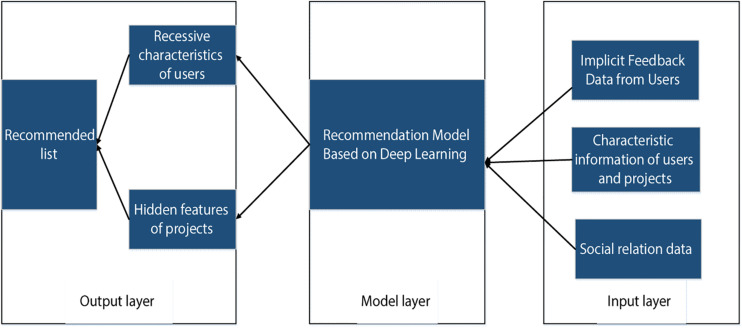
Schematic diagram of traditional deep learning recommendation.

Recommendation system refers to the process of using data mining technology and a prediction algorithm to predict the information or commodities that users are interested in according to a large number of users’ behavior data, project feature data, user feature data and so on. As shown in Figure 2, the structure of traditional deep learning recommendation systems includes input layer module, model layer module, and output layer module. The input layer and output layer modules are basically the same as the traditional recommendation algorithm, but there are some differences in the model layer. The models adopted by the deep learning recommendation algorithm framework include convolutional neural network, deep neural network, self-encoder, cyclic neural network, deep confidence network, etc., that is, the methods of extracting features are different (Shen et al., 2019). The traditional deep learning recommendation technology applies the powerful ability of feature extraction to extract more meaningful features from the low-level features, and thus indicate the effectiveness of the recommendation results.

### A Deep Neural Network Algorithm Model Based on PMF

In order to optimize the deep neural network recommendation algorithm, the PMF algorithm (Jiang et al., 2019) was introduced. This algorithm could decompose latent factor of user and item by matrix, and then connect user interest and item by latent factor. Assuming that the number of users is N and the number of items is M, the scoring matrix can be expressed as*R* = *R*^*N*×*M*^. Then, the implicit feature matrix of users and projects is obtained through matrix *R* operation.

(1) $$\begin{array}{c} U=R^{k\times N}\\ V=R^{k\times M} \end{array}$$

Where U represents the implicit characteristics of the user; V represents the implied characteristics of the item. Assuming that the implicit feature vectors of users and items follow a normal distribution, the expression is as follows.

(2) $$P\,\left(U|\sigma_{U}^{2}\right)=\prod_{i=1}^{N}N\,\left(u_{i}|\sigma_{U}^{2}\,I\right)$$

(3) $$P\,\left(V|\sigma_{V}^{2}\right)=\prod_{j=1}^{N}N\,\left(v_{i}|\sigma_{U}^{2}\,I\right)$$

Where *N*(*x*|μ,σ^2^) is the probability density function of the normal distribution, μ is the mean, σ^2^ is the variance; u_*i*_ represents the implicit eigenvector of the user; v_*j*_ represents the implicit feature vector of the project; I is the identity matrix.

In the deep neural network algorithm model based on PMF in this research, the implicit feature V of the project is obtained by introducing the deep neural network model operation. Suppose that the parameter set of the deep neural network is W and follows a normal distribution, then the equation below is obtained.

(4) $$V_{j}=d\,n\,n\,\left(W,X_{j}\right)+\varepsilon_{j}$$

Where *dnn*() represents the deep neural network; ε_*j*_ represents the random error and follows the normal distribution, which can be expressed as follows.

(5) $$\varepsilon_{j}∼N\,\left(0,\sigma_{v}^{2}\,I\right)$$

The parameter set W of the deep neural network is set to follow a normal distribution, then the expression equation is as follows.

(6) P⁢(W|σW2)=∏k=1pN⁢(Wk|0,σ2W)

Where *N*(*x*|μ,σ^2^) is the probability density function of the normal distribution, σ^2^ is the variance, *W*_*k*_ represents the implicit eigenvector of the parameter set of the depth neural network. Therefore, the conditional probability of implicit feature V of the project is as follows.

(7) P⁢(V|W,X,σV2)=∏j=1mN⁢(Vj|d⁢n⁢n⁢(W,Xj),σ2V⁢I)

Where *dnn*() represents the deep neural network, I is the identity matrix, σ^2^ is the variance; then the probability distribution of V, U, and W can be calculated by introducing the Bayesian equation.

$$P\,\left(U,V,W|R,X,\sigma^{2},\sigma_{U}^{2},\sigma_{V}^{2},\sigma_{W}^{2}\right)=$$

(8) $$P\,\left(R|U,V,\sigma^{2}\right)\,P\,\left(U|\sigma_{U}^{2}\right)\,P\,\left(V|W,X,\sigma_{V}^{2}\right)\,P\,\left(W|\sigma_{W}^{2}\right)$$

In this way, a deep neural network algorithm model based on PMF is constructed.

### An Entrepreneurial Project Recommendation Algorithm Based on Optimized Deep Neural Network

**FIGURE 3:**
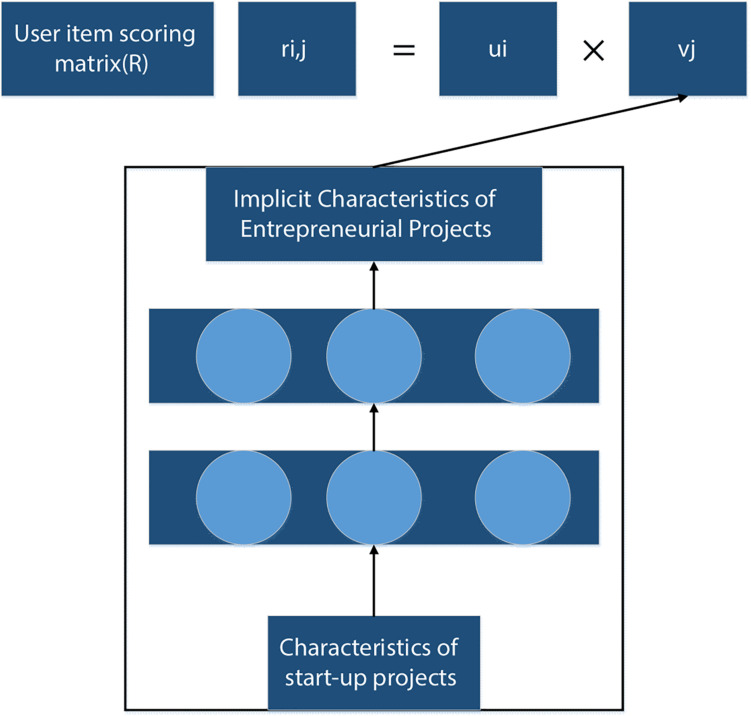
Schematic diagram of recommendation algorithm structure of entrepreneurial project based on optimized deep neural network.

According to the above optimization algorithm steps, the algorithm model structure of a deep neural network based on PMF is shown in Figures 2, 3, which mainly includes the PMF module and the deep neural network module. The PMF module is used to obtain the hidden characteristics of users, and the deep neural network module inputs the characteristics of entrepreneurship project to output the hidden characteristics of the project.

**FIGURE 4:**
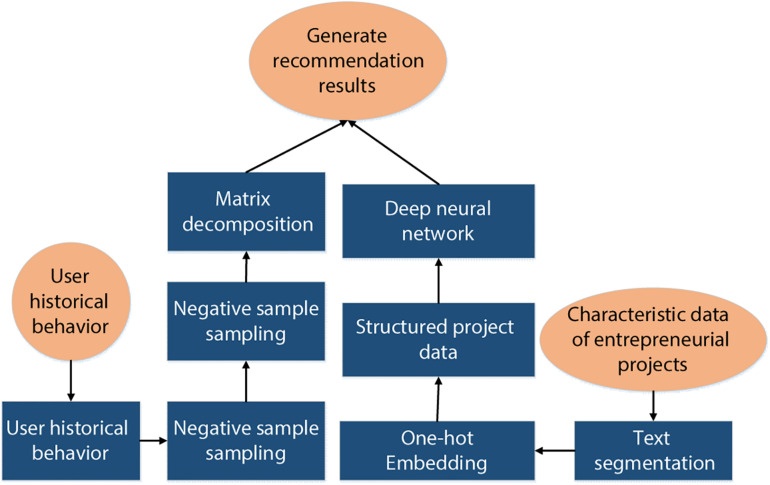
Flow diagram of entrepreneurial project recommendation algorithm.

Figure 4 is a schematic diagram of the process of the recommendation system for entrepreneurial projects. There are many characteristics of entrepreneurial projects, such as text information, labels of entrepreneurial projects, involved platforms, time, and financing rounds. In order to focus this information on the recommendation system analysis, the recommendation process is divided into the following parts.

#### Analysis of User Historical Behavior Data

According to the historical behavior information of the users in the entrepreneurial project, the score data of the users on the entrepreneurial project was calculated, and the negative sample sampling was carried out to generate the score matrix. The degree of users’ interest in an entrepreneurial project was mainly analyzed from the two dimensions of interaction times and interaction behaviors. The more interaction times, the higher the user’s interest degree, while the interactive behaviors such as thumb up, favorites, and comments can reflect the increasing degree of users’ interest. The user’s score of the project can be expressed in the following equation.

(9) $$r_{u\,i}=\sum_{j=1}^{k}a_{j}\,q_{u\,i,j}$$

where, *r*_*ui*_ is how users rate a start-up project, *a*_*j*_ is the weight of the *j*th interaction dimension, *q*_*ui,j*_ represents the number of times a user interacts with a start-up project in the *j*th interaction dimension.

#### Application of Recommendation Algorithm Model

The recommendation algorithm of entrepreneurial project based on optimized deep neural network was used to obtain the hidden characteristics of the entrepreneurial project from the text information and structured content information of the project, and the learning training of the model was carried out.

#### Generation of the Recommendation Results

The trained model was used to generate recommendations for users.

### The Theory of Personality Development

Human personality development refers to the formation and development of human personality from birth to adolescence, which is also the key theoretical basis for colleges to conduct entrepreneurship psychology education. The entrepreneurship education should be people-oriented, and full consideration should be given to each student’s personality and to creating a harmonious teaching environment, so that individual differences between students can be understood. In the design of entrepreneurship courses, the students’ needs, interests and goals should be respected. Besides, different methods should be adopted for different students, so as to guide students to study entrepreneurship courses and achieve the purpose for setting up entrepreneurship psychological education courses in colleges (Shen and Ho, 2020).

### The Evaluation Criteria of the Recommendation Algorithm

After designing the recommendation algorithm, the researchers often need to evaluate the performance of the algorithm. Currently, the common evaluation indexes include MSE, recall rate and accuracy (Dasgupta and Ghosh, 2019).

#### MSE

The MSE is mainly used to evaluate the error between the predicted score and the real score. The calculation equation is as follows.

(10) $$M\,S\,E=\sqrt{\frac{1}{\left|T\right|}\,\sum_{i=1}^{\left|T\right|}{\left(r_{i}-\hat{r_{i}}\right)}^{2}}$$

Where T represents the test set, |*T*| Represents the number of test sets; *r*_*i*_ is the true score; $\hat{r_{i}}$ is the prediction score.

#### Recall Rate and Accuracy

Recall rate and accuracy were used to evaluate the Top-k problem. If there is no relevant rating information, the recall rate and accuracy rate should be used to evaluate the performance of the recommendation system when only user browsing information is known. Then the calculation equation of recall rate and accuracy can be as follows.

(11) $$\mathrm{Precision}=\frac{T\,P}{T\,P+F\,P}$$

(12) $$\mathrm{Recall}=\frac{T\,P}{T\,P+F\,N}$$

Where TP Represents a positive sample of the true category; FP Represents a positive sample of the predicted category; FN represents a negative sample of the predicted category.

### Design of Questionnaire

The questionnaire survey is a statistical analysis method often used in social science research. It is usually used to measure people’s characteristics, attitudes or behaviors. In this study, college students’ entrepreneurial intention questionnaire and college students’ entrepreneurial mental resilience scale were selected to evaluate the intervention effect before and after entrepreneurship education.

#### Questionnaire of College Students’ Entrepreneurial Intention

The questionnaire includes two dimensions – entrepreneurial possibility and entrepreneurial behavior tendency. Entrepreneurial possibility includes two measurement items, and entrepreneurial behavior tendency includes five measurement items. The subjects were asked to score for each item from one point to five points, with one point indicating that they are very inconsistent and five points indicating that they are very consistent. The score range of entrepreneurial possibility is 2–10, and the score range of entrepreneurial behavioral tendency is 5–25. The higher the score, the stronger the entrepreneurial intention, see Table 1.

**TABLE 1:** Questionnaire of college students’ entrepreneurial intention.

**Variables**	**Measuring item**
Entrepreneurial possibilities	I have a good chance of starting my own business within 5 years of graduation. There’s a good chance I’ll start my own business one day
Entrepreneurial behavior tendency	I’ve thought about business plans or plans I tried to raise money for my future company I took the initiative to understand the detailed process of starting a company I’ve thought about industries and projects I’d like to start in the future I thought about starting a start-up team

#### College Students’ Entrepreneurial Mental Resilience Scale

The scale includes three dimensions – entrepreneurial resilience, entrepreneurial strength, and optimism. Entrepreneurial resilience includes four items, entrepreneurial strength with four items, and optimism with three items. The subjects were asked to score for each item from one point to five points, with one point indicating that they are very inconsistent and five points indicating that they are very consistent. The score ranges from 4 to 20 for entrepreneurial resilience, 4 to 20 for entrepreneurial strength, and 3 to 15 for optimism. The higher the score, the stronger the entrepreneurial intention, see Table 2.

**TABLE 2:** College students’ entrepreneurial mental resilience scale.

**Variables**	**Measuring item**
Entrepreneurial tenacity	I will not give up even if I encounter difficulties in starting a business. I believe I can achieve my goals I’d rather take the lead on my own rather than let someone else make the decision I am a person who likes to challenge difficulties I am a person who will be very strong in the face of difficulties
Entrepreneurial strength	When the entrepreneurial environment changes, I think I can adapt The difficulties I have experienced in the past have made me stronger No matter what the result, I will continue to work hard No matter what difficulties I encounter in starting a business, I will try my best to achieve my goal
Optimism	In the face of entrepreneurial difficulties, I believe that at least one partner will help me I believe I can handle whatever comes my way when starting a business I try to get started on the bright side of trouble when my business is facing a setback

SPSS19.0 statistical software was used to analyze the data in this study. *T* test was used to compare the data between the two groups, with Origin8.0 mapping.

## Results

### Performance Analysis of Entrepreneurial Project Recommendation Algorithm Based on Optimized Deep Neural Network

This test was conducted on a real data platform. The operating system was: ubuntu 16.04 standard edition 64-bit; CPU for Intel Xeon E5-2682V4; the 32 Gb memory; the hard disk was 500G solid state disk; the development language was Python; the deep learning framework was TensorFlow. Currently, there is no public data set that can be used for entrepreneurship projects recommendation, so the Python crawler is adopted to crawl data from the entrepreneurship website platform to obtain the behavioral data of users, the content characteristic information of entrepreneurial projects, the content characteristic information of users, etc., and the standardized processing was carried out for the following tests.

In order to intuitively demonstrate the performance of the algorithm in this research, the PMF algorithm (Hendrickx et al., 2020) and the DBN algorithm (Zhang et al., 2019) were introduced for comparative analysis.

Test: firstly, the number of items recommended for a user was set as 60, 120, 180, 240, and 300, and the remaining conditions remained the same. The platform ran three algorithms and calculated the recall rate, accuracy rate and MSE of the three algorithms.

**FIGURE 5:**
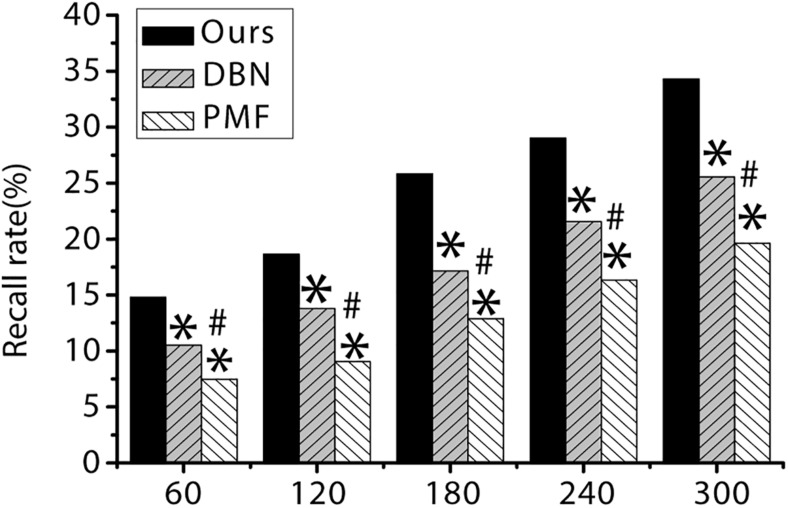
The recall rate of three algorithms under different item quantity. ^∗^ indicates that the difference is statistically significant compared with the algorithm proposed in the study (*p* < 0.05). ^#^ indicates that the difference is statistically significant compared with DBN algorithm (*p* < 0.05).

**FIGURE 6:**
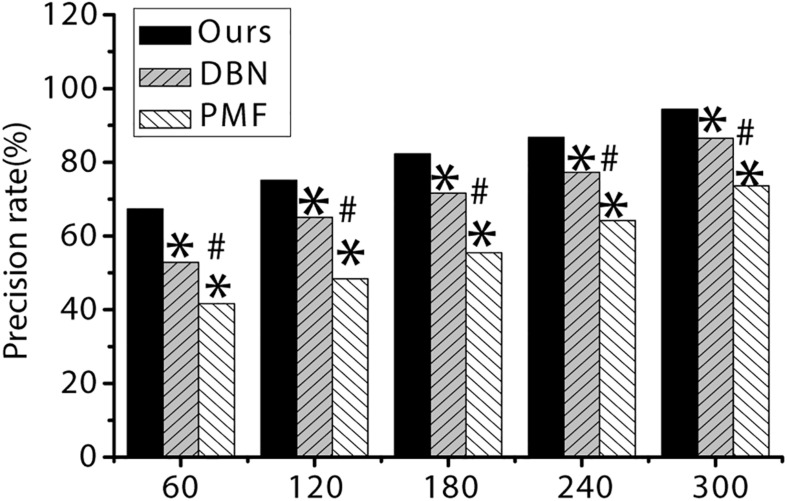
The accuracy of the three algorithms under different item quantity. ^∗^ indicates that the difference is statistically significant compared with the algorithm proposed in the study (*p* < 0.05). ^#^ indicates that the difference is statistically significant compared with DBN algorithm (*p* < 0.05).

As shown in Table 3 and Figures 5, 6 below, the recall rate and accuracy of the algorithm proposed in the study are always significantly higher than the PMF algorithm and the DBN algorithm when the number of items is 60, 120, 180, 240, and 300, and the difference is statistically significant (*p* < 0.05).

**TABLE 3:** Recall rate and accuracy of three algorithms with different number of items.

**Number**	**PMF algorithm**		**The proposed algorithm**		**DBN algorithm**	
			
	**Recall rate**	**Accuracy rate**	**Recall rate**	**Accuracy rate**	**Recall rate**	**Accuracy rate**
60	7.47%	41.66%	14.81%	67.35%	10.51%	52.81%
120	9.05%	48.41%	18.63%	75.16%	13.78%	65.06%
180	12.90%	55.42%	25.82%	82.27%	17.17%	71.64%
240	16.33%	64.21%	29.01%	86.81%	21.57%	77.31%
300	19.64%	73.65%	34.29%	94.37%	25.55%	86.53%

**FIGURE 7:**
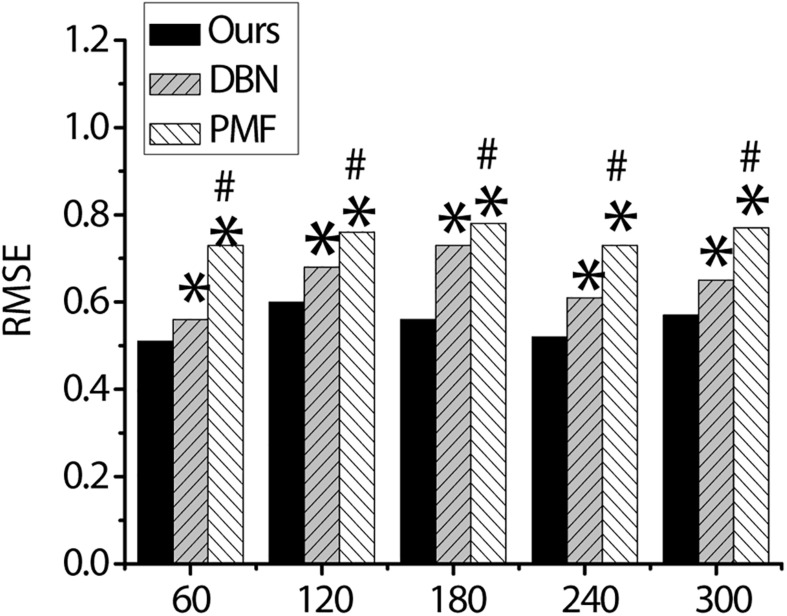
The MSE of the three algorithms. ^∗^ indicates that the difference is statistically significant compared with the algorithm proposed in the study (*p* < 0.05). ^#^ indicates that the difference is statistically significant compared with DBN algorithm (*p* < 0.05).

Figure 7 shows RMSE of the proposed algorithm is always significantly lower than that of the PMF algorithm and the DBN algorithm when the number of items is 60, 120, 180, 240, and 300, and the difference is statistically significant (*p* < 0.05).

### Comparison of Entrepreneurial Intention Before and After Entrepreneurial Psychological Education Training Between the Experimental Group and the Control Group

**FIGURE 8:**
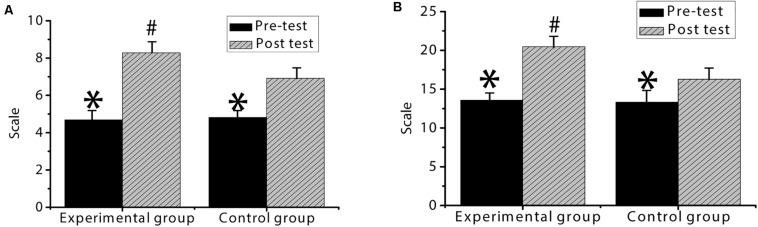
Comparison of entrepreneurial intention between the experimental group and the control group. **(A)** is the entrepreneurial possibility of the two groups of students; **(B)** is the entrepreneurial behavior tendency of the two groups. ^∗^ indicates that the difference is statistically significant compared with the post-test entrepreneurial intention (*p* < 0.05); ^#^ indicates that the difference is statistically significant compared to the control group (*p* < 0.05).

As shown in Figure 8, the pre-test score of the experimental group is 4.69 ± 0.76, and the post-test score of the experimental group is 8.28 ± 1.52. The pre-test score of entrepreneurial behavior tendency is 13.56 ± 3.28, and the post-test score of entrepreneurial behavior tendency is 20.47 ± 6.14. In the control group, the pre-test score of entrepreneurial possibility is 4.81 ± 0.83, and the post-test score of entrepreneurial possibility is 6.92 ± 0.86. The pre-test score of entrepreneurial behavior tendency is 13.32 ± 4.11, and the post-test score of entrepreneurial behavior tendency is 16.29 ± 5.07. There is no statistically significant difference between the pre-test scores of the experimental group and the control group (*p* > 0.05). The post-test scores of entrepreneurial possibilities and the entrepreneurial behavior tendency of the two groups are significantly higher than the pre-test scores, and the difference is statistically significant (*p* < 0.05). The score of entrepreneurial possibility and entrepreneurial behavior tendency of the experimental group is significantly higher than that of the control group, and the difference is statistically significant (*p* < 0.05).

### Comparison of Mental Resilience Between the Experimental Group and the Control Group Before and After Entrepreneurial Psychological Education and Training

**FIGURE 9:**
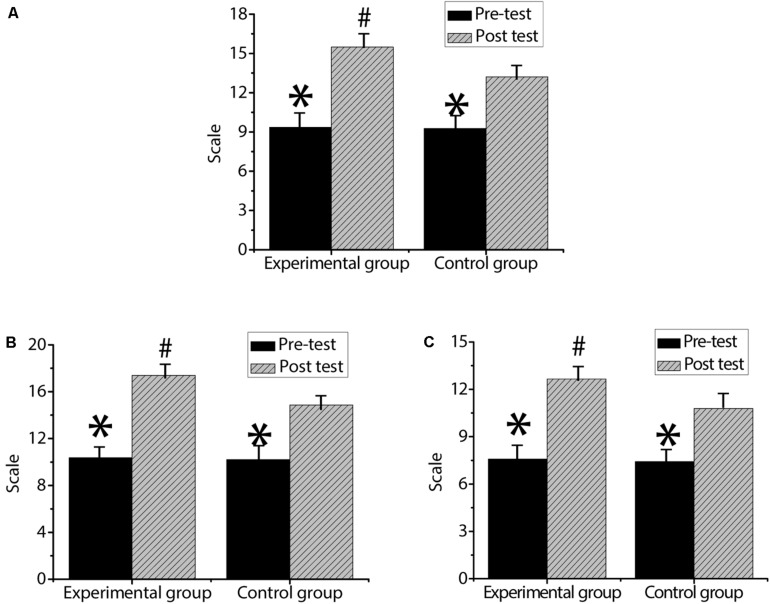
Comparison of mental resilience between the experimental group and the control group. **(A)** is the entrepreneurial resilience of the two groups of students; **(B)** is the entrepreneurial strength of the two groups of students; **(C)** is the optimism dimension of the two groups of students. ^∗^ indicates that the difference is statistically significant compared with the post-test entrepreneurial intention (*p* < 0.05); ^#^ indicates that the difference is statistically significant compared to the control group (*p* < 0.05).

As shown in Figure 9, the score of entrepreneurial tenacity in the experimental group is 9.36 ± 0.64 in the pre-test and 15.49 ± 3.44 in the post-test. The pre-test score of entrepreneurial strength is 10.37 ± 2.46, and the post-test score of entrepreneurial strength is 17.40 ± 7.23. The score of the optimism dimension is 7.58 ± 1.12 in the pre-test and 12.66 ± 4.03 in the post-test. In the control group, the pre-test score of entrepreneurial tenacity is 9.27 ± 0.59, and the post-test score of entrepreneurial tenacity is 13.21 ± 2.26. The pre-test score of entrepreneurial strength is 10.21 ± 2.08, and the post-test score of entrepreneurial strength is 14.87 ± 5.16. The score of the optimism dimension is 7.42 ± 0.78 in the pre-test and 10.79 ± 5.72 in the post-test. Among them, there is no statistically significant difference between the experimental group and the control group in the dimensions of entrepreneurial tenacity, entrepreneurial strength, and optimism in the pre-test (*p* > 0.05). The scores of entrepreneurial tenacity, entrepreneurial strength, and optimism in the post-test of the two groups are significantly higher than the pre-test scores, and the difference is statistically significant (*p* < 0.05). The scores of entrepreneurial tenacity, entrepreneurial strength and optimism in the experimental group are significantly higher than those in the control group, and the differences are statistically significant (*p* < 0.05).

## Discussion

In recent years, with the development of computer networks and social networks, the dissemination of network information has been greatly improved, which provides convenience for more users to obtain information. However, serious information overload will occur when there is a huge amount of information, and the recommendation algorithm is the proposed solution to this problem (Bux and van Vuuren, 2019; Fuster et al., 2019). In this study, the PMF algorithm was firstly introduced to optimize the deep neural network algorithm model. Then, according to the characteristics of the entrepreneurial project, the recommendation algorithm of the optimized deep neural network was established, and the simulation experiment was analyzed. The results show that the system recall rate and accuracy based on the proposed algorithm are always significantly higher than the PMF algorithm and the DBN algorithm, and the difference is statistically significant (*p* < 0.05); the MSE of the proposed algorithm is significantly smaller than that of the PMF algorithm and the DBN algorithm, and the difference is statistically significant (*p* < 0.05), which is basically consistent with the research results of Zulfiqar et al. (2019), indicating that the proposed algorithm has better operational accuracy and performance as compared with the traditional algorithm, and the incorporation of the content and feature information of entrepreneurial projects into the recommendation system can significantly improve the performance of the recommendation algorithm (Etemad, 2019; McAdam et al., 2019). This is mainly due to the fact that the data information of the project can feedback the users’ selection preference within a certain range, that is, users in different regions may only pay attention to the local entrepreneurial information (Rippa and Secundo, 2019).

At present, many college students actually have the idea of their own businesses after graduation, but their entrepreneurial ability are relatively weak due to the lack of social experience. They have no clear plan for their future entrepreneurial behavior; simply speaking, they are confused about their suitable entrepreneurial direction, and the courses of entrepreneurship psychology education in universities are often at the theoretical level (Badri and Hachicha, 2019; Hemmert et al., 2019). Accordingly, the entrepreneurship project recommendation algorithm based on the optimization of deep neural network proposed in this research is applied to the courses of entrepreneurship psychology education and training to help college students clearly choose the direction of self-employment, and the students’ entrepreneurial intention and entrepreneurial mental resilience before and after the training are compared. Firstly, there is no statistically significant difference between the two groups in the scores of the dimensions of entrepreneurial possibility, entrepreneurial behavior tendency, entrepreneurial tenacity, entrepreneurial strength, and optimism (*p* > 0.05), which shows that the two groups of students are homogenous. In addition, it is found that the scores of entrepreneurial tenacity, entrepreneurial strength, and optimism dimension in the post-test of the two groups are significantly higher than the pre-test scores, but the post-test scores of the experimental group are significantly higher than those of the control group (*p* < 0.05), which is consistent with the research results of Passoni et al. (2019), indicating that the recommendation algorithm for entrepreneurship projects based on the optimization of deep neural network can effectively solve some problems that contemporary college students will face when starting their own businesses, and improve their entrepreneurial intention and psychological resilience. Therefore, courses of entrepreneurship education for college students should focus on practical solutions to students’ entrepreneurship problems, help students clarify the direction of entrepreneurship, and combine theory with practice to improve the effectiveness of entrepreneurship education.

## Conclusion

In this study, the PMF algorithm was introduced to optimize the deep neural network algorithm model. Then, based on the personality development theory, a recommendation system for entrepreneurial projects based on the optimized deep neural network was established, which was applied to the practical entrepreneurship psychology education courses while the simulation experiment analysis was conducted, thereby providing experimental basis for the practicability of entrepreneurship psychology education courses for college students in the future. However, there are still some problems to be solved in this study. The selected students only come from a certain region, leading to certain regional limitations and the constrained popularization of the research results. In addition to the entrepreneurship data information in general entrepreneurship website, there are news data and other information. In the future, cross-domain information may be introduced into the recommendation system for research. In a word, compared with the traditional system, the recommendation algorithm for entrepreneurial projects based on the theory of personality development and the optimized deep neural network has better performance, so that it can effectively solve contemporary college students’ entrepreneurship psychological problems and improve their entrepreneurial intention and psychological resilience.

## Data Availability Statement

The datasets generated for this study are not publicly available due to restrictions set by Northeastern University committee. Requests to access these datasets should be directed to zhenchenneu@163.com.

## Ethics Statement

The studies involving human participants were reviewed and approved by the Northeastern University Ethics Committee. The patients/participants provided their written informed consent to participate in this study.

## Author Contributions

ZC wrote the manuscript. XY made the validation. Both authors contributed to the article and approved the submitted version.

## Conflict of Interest

The authors declare that the research was conducted in the absence of any commercial or financial relationships that could be construed as a potential conflict of interest.
